# Acyl-CoA thioesterases and their derived lipid mediators: linking Acyl-CoA metabolic reprogramming to the tumor microenvironment

**DOI:** 10.3389/fimmu.2026.1917412

**Published:** 2026-09-09

**Authors:** Yimeng Ren, Qingjun Wang, Xiujuan Qu, Xiaofang Che

**Affiliations:** 1 Department of Medical Oncology, the First Hospital of China Medical University, Shenyang, China; 2 Key Laboratory of Anticancer Drugs and Biotherapy of Liaoning Province, the First Hospital of China Medical University, Shenyang, China; 3 Department of Oncology, The First Affiliated Hospital of Jinzhou Medical University, Jinzhou, China

**Keywords:** Acyl-CoA thioesterase, fatty acids, lipid mediators, metabolic reprogramming, tumor microenvironment

## Abstract

Metabolic reprogramming in tumors is a fundamental mechanism by which tumor cells adapt to proliferative stress and evade immune surveillance. In recent years, the regulatory role of lipid metabolism—particularly acyl-CoA metabolism—within the tumor microenvironment has garnered increasing attention. As a central hub of lipid metabolism, acyl-CoA homeostasis directly determines cellular energy status, membrane lipid composition, and signal output. The acyl-CoA thioesterase (ACOT) family regulates intracellular homeostasis of free fatty acids (FFAs) and acyl-CoA by hydrolyzing the thioester bond of acyl-CoA, thereby influencing membrane lipid composition, ferroptosis sensitivity, and immune cell function, and consequently promoting tumor progression, drug resistance, and microenvironmental remodeling. In this context, this review systematically summarizes the structural and functional characteristics of the ACOT family, elucidates the regulatory networks of its derived lipid mediators in tumors, and describes its effects on tumor metabolism and the microenvironment through interactions with other metabolic enzymes. This review aims to reveal the pivotal role of the ACOT family in the tumor metabolism-immunity network, update our understanding of its complex roles in tumors, and provide theoretical insights and future research directions for its potential as a drug target and for overcoming treatment resistance.

## 1 Introduction

Cancer cells are characterized by their eximious ability to adapt and survive within harsh microenvironments, including poor oxygenation and nutrient deprivation (1). Compared to normal tissues, the tumor microenvironment (TME) exhibits hyperactive metabolic networks, driven by oncogenic signals that activate multiple anabolic pathways, such as glycolysis, one-carbon metabolism, the tricarboxylic acid (TCA) cycle, and fatty acid synthesis. These diverse metabolic pathways are essential not only for cancer cell proliferation but also for normal proliferating cells, including immune cells (2). As a result, metabolic reprogramming has been established as a critical link between tumor progression and immune evasion. Among these metabolic alterations, the hyperactivity of lipid metabolism holds particular significance: tumor cells enhance fatty acid uptake and synthesis to meet the energy and membrane synthesis demands of rapid proliferation, while infiltrating immune cells depend on fatty acid oxidation to sustain their effector functions and longevity (3).

Acyl-CoA metabolism serves as a central hub in the lipid metabolism network. Under normal physiological conditions, acyl-CoA acts as a convergence point for the metabolism of carbohydrates, fats, and amino acids—the three major nutrient categories. Pyruvate, produced from glucose via glycolysis, enters the mitochondria and is converted into acetyl-CoA; fatty acids undergo β-oxidation to produce acyl-CoA molecules of varying chain lengths; and the carbon skeletons of certain amino acids can also be converted into acetyl-CoA or acyl-CoA (4). Acyl-CoA derived from these sources integrates into a shared metabolic pool, utilized for β-oxidation to generate energy, the synthesis of membrane phospholipids, and post-translational modifications of proteins (such as acetylation and palmitoylation) (5). These modifications play key roles in non-metabolic processes such as enzyme activity regulation, ion channel function, membrane transport, and transcriptional regulation. However, the maintenance of the acyl-CoA pool is not maintained spontaneously; instead, it relies on a delicate balance among synthesis, utilization, and hydrolysis pathways. The synthesis pathway is catalyzed by the ACSL family, which converts free fatty acids into acyl-CoA (6), while the utilization pathway is mediated by CPT1 and CPT2, which facilitate mitochondrial transport and β-oxidation of acyl-CoA (7). In contrast, the hydrolysis pathway is governed by the Acyl-CoA Thioesterases (ACOTs), which release free fatty acids and CoA through the hydrolysis of thioester bonds.

Tumor cells frequently reprogram acyl-CoA metabolism by altering the expression and metabolic flux of key enzymes across these three pathways to cope with proliferative and oxidative stress (8). At the synthetic end, the ACSL family is typically upregulated, enhancing fatty acid activation and esterification to provide substrates for tumor cell membrane synthesis and signal transduction. At the hydrolytic end, multiple ACOT family members (such as ACOT7, ACOT8, and ACOT11) are also upregulated, accelerating the hydrolysis of specific acyl-CoA species, reshaping the free fatty acid profile and membrane lipid composition, and altering post-translational modifications, thereby influencing tumor cell proliferation, survival, and metabolic adaptation. However, compared with the extensive research on the ACSL family at the synthetic end (9, 10), systematic summaries of the ACOT family at the hydrolytic end remain relatively scattered and lack comprehensive integration.

Recognizing the central role of the ACOTs and their derived-metabolites in lipid metabolic reprogramming within the tumor microenvironment, this review systematically examines their regulatory mechanisms and therapeutic potential. It begins by addressing the role of the ACOTs within the broader acyl-CoA metabolic network, highlighting substrate preferences—including chain-length selectivity and saturation specificity—and explaining how subcellular compartmentalization confers distinct functional roles to different ACOT members. Building on this foundation, the review examines how ACOTs influence downstream signaling profiles by modulating the dynamic equilibrium of the acyl-CoA pool. Particular attention is given to free fatty acids and acyl-CoA, which act as lipid signaling molecules mediating interactions between tumor and immune cells, thereby translating metabolic states into immune-regulatory signals within the tumor microenvironment. Lastly, the review discusses the therapeutic potential of targeting ACOTs as part of combination strategies, offering a mechanistic framework and theoretical foundation for advancing translational research in tumor metabolism and immune crosstalk (11).

## 2 ACOTs in the acyl-CoA metabolic network

### 2.1 ACOTs mediates the dynamic balance of the Acyl-CoA pool

The dynamic equilibrium of acyl-CoA intracellular pool affects metabolic crosstalk between tumor cells and immune cells. When tumor cells alter the expression of enzymes involved in acyl-CoA metabolism, the disrupted equilibrium of acyl-CoA in the tumor microenvironment reshapes the functional phenotypes of immune cells. Research shows that abnormally high levels of acyl-CoA metabolism in tumor cells can deplete free fatty acids in the microenvironment, leading to metabolic exhaustion in nearby CD8^+^ T cells due to a lack of substrates for fatty acid oxidation. This results in the loss of their effector function, thereby creating an immunosuppressive microenvironment driven by metabolic competition (12). Thus, precise regulation of key enzymes in acyl-CoA metabolism represents a crucial strategy for manipulating the metabolic-immune dialogue within the tumor microenvironment.

As key executors of acyl-CoA hydrolysis, the ACOT family influences both energy metabolism and biosynthesis by regulating the availability of acetyl-CoA and other acyl-CoA molecules. Additionally, they modulate protein acetylation and downstream signaling pathways, thereby affecting the metabolic adaptation and immune evasion of tumor cell (13). From the perspective of metabolic regulation, the cycle of acyl-CoA hydrolysis and resynthesis catalyzed by ACOT shares similarities with the classic Warburg effect observed in tumor cells—both appear to be “seemingly inefficient energy wastage”, yet in reality serve higher-order biological functions. In the Warburg effect, tumor cells predominantly rely on glycolysis even under aerobic conditions. However, this “inefficiency” is not a metabolic defect but a trade-off: it sacrifices energy yield to ensure a rapid supply of glycolytic intermediates, which are critical for the biosynthesis of nucleotides, amino acids, and lipids, thereby supporting the rapid proliferation of tumor cells (14). Indeed, the Warburg effect not only supports biosynthesis but also enhances tumor aggressiveness and therapeutic resistance through multiple mechanisms (15). Similarly, ACOTs catalyze the hydrolysis of acyl-CoA into free fatty acids and CoA, which are subsequently re-esterified by ACSL to regenerate acyl-CoA. Although ATP is initially consumed to synthesize acyl-CoA (fatty acid + CoA + ATP → acyl-CoA + AMP + 2Pi), the hydrolysis reaction catalyzed by ACOTs break the high-energy thioester bond, dissipating stored energy as heat and creating a seemingly “energy-wasting” futile cycle. Despite this apparent inefficiency, the process has crucial regulatory functions: First, the hydrolysis reaction liberates free CoA, preventing mitochondrial β-oxidation bottlenecks caused by CoA depletion (16); Second, when energy demand decreases or acyl-CoA accumulates excessively, ACOTs rapidly clear surplus metabolites, mitigating the lipotoxicity associated with long-chain acyl-CoA. Through this “hydrolysis-resynthesis” cycle, ACOTs act as a critical regulatory node, maintaining the size of the intracellular acyl-CoA pool and controlling the flow of fatty acids within the cell.

**Figure 1:**
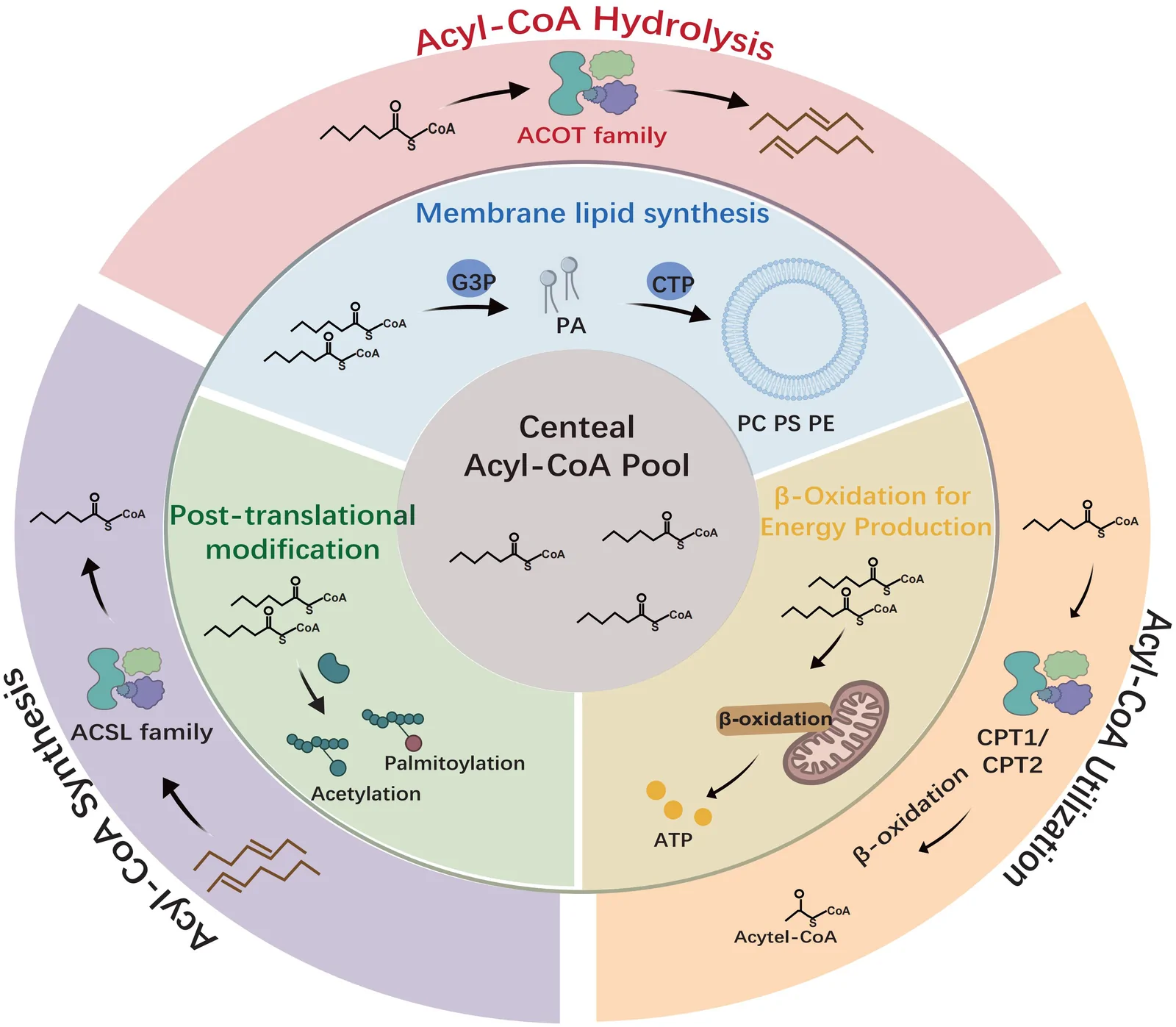
Acyl-CoA is synthesized by ACSL, utilized by CPT1-mediated β-oxidation, and hydrolyzed by ACOTs. As the dedicated hydrolysis arm, ACOTs control acyl-CoA pool homeostasis and determine lipid flux toward energy production, membrane synthesis, and post-translational modification (PTMs). Created in BioRender. Ren. (2026) https://BioRender.com/z2i12hd.

In recent years, there has been a surge of research on the metabolic reprogramming of acyl-CoA (17). Taking ACSL4 as an example, this enzyme catalyzes the conversion of free fatty acids into polyunsaturated acyl-CoA, which is subsequently incorporated into membrane phospholipids. This process reshapes membrane lipid composition, enhances cancer cell membrane fluidity, and promotes vascular extravasation and metastatic colonization (18). Although ACOTs play equally significant roles as ‘down regulators’ of the acyl-CoA pool by influencing its dynamic balance, their biological significance has not received comparable attention. Consequently, systematically unraveling the functions of ACOT-mediated hydrolysis pathways within the acyl-CoA metabolic network, especially in the context of the tumor microenvironment, holds substantial theoretical and translational importance (**Figure 1**).

### 2.2 The catalytic mechanism and substrate specificity of ACOTs

The human ACOT family consists of 12 members: 10 ACOT proteins and two homologous proteins, THEM4 and THEM5 (also known as CTMP1 and CTMP2). Importantly, ACOT family members exhibit distinct subcellular compartmentalization and substrate preferences. Chemically, ACOT catalyzes the hydrolytic cleavage of thioester bonds (R-C(=O)-S-CoA), producing free fatty acids (R-COOH) and reduced coenzyme A (HS-CoA) (19).

Although members of ACOTs catalyze the same reaction, they do not share similar structures or sequence homology. Based on their structural features, ACOTs are categorized into two major groups: Type I ACOTs (ACOT1, ACOT2, ACOT4, ACOT6) belong to the α/β-hydrolase superfamily and possess a typical Ser-His-Asp catalytic triad; Type II ACOTs (ACOT7, ACOT8, ACOT9, ACOT11, ACOT12, ACOT13, THEM4, THEM5) belong to the HotDog superfamily, characterized by a unique conformation in which a curved antiparallel β-sheet wraps around an α-helix. Notably, Type II ACOTs typically require dimer or tetramer formation to create a complete active site (20). The dimeric forms of Type II ACOTs can vary: some consist of two HotDog domains (e.g., ACOT7–12), while others involve two oligomers within a single HotDog domain (e.g., ACOT13, THEM4, THEM5) (21). Additionally, ACOT11 and ACOT12 possess a lipid-binding C-terminal Steroidogenesis Acute Regulatory Transfer-associated (START) domain. As a result, they are also classified as StarD14 and StarD15 within the START domain protein family (22). This structural diversity across ACOTs contribute to differences in substrate preferences and catalytic efficiencies among its members.

Different ACOTs exhibit distinct substrate preferences. ACOT12 predominantly hydrolyzes short-chain acyl-CoA substrates (C2–C4), with a particular affinity for acetyl-CoA (23); ACOT7 exhibits activity toward medium-chain acyl-CoA (C8-C12) (24);Members localized in the peroxisome, such as ACOT2, ACOT44, and ACOT12—prefer long-chain acyl-CoA substrates (C14–C18), with ACOT11 displaying the highest activity for palmitoyl-CoA and myristoyl-CoA (25); Conversely, ACOT8 and ACOT9 function as broad-spectrum enzymes, exhibiting significant hydrolytic activity toward both short-chain and long-chain acyl-CoA substrates (26). The molecular basis for the precise recognition of substrates with varying chain lengths is rooted in differences in the depth and geometric conformation of the hydrophobic pocket within the enzyme’s active site. The depth, width, and spatial arrangement of internal amino acid residues collectively create a physical barrier, functioning as a strict “filter” to select substrate fatty acid chains based on their length and degree of unsaturation (27).

The substrate selectivity of ACOTs extends beyond carbon chain length, as it also distinguishes between the degrees of fatty acid saturation. For instance, ACOT8 and ACOT7 exhibit a pronounced preference for polyunsaturated fatty acid-CoA (PUFA-CoA) substrates, such as arachidonyl-CoA (C20:4) and linoleoyl-CoA (C18:2) (24). In contrast, ACOT1 and ACOT2 display higher activity toward saturated and monounsaturated acyl-CoA substrates (23). This substrate specificity directly impacts the phospholipid composition of cell membranes and their susceptibility to lipid peroxidation (28). For example, ACOT7 preferentially hydrolyzes PUFA-CoA, thereby influencing the saturation of fatty acids entering the membrane phospholipid synthesis pathway. This process reduces the incorporation of polyunsaturated fatty acids (PUFAs) into membrane lipids, thereby limiting their availability as substrates for lipid peroxidation. As a result, under oxidative stress conditions, a metabolic barrier against ferroptosis is established, which subsequently promotes chemoresistance (29). In TME, alterations in lipid saturation also significantly impact immune responses. Changes in membrane lipid composition regulate the localization of immune checkpoint molecules, such as PD-L1 (30), while shifts in PUFA metabolic flux influence macrophage polarization and T-cell function by modulating the production of immunomodulatory mediators, including prostaglandins (31). Consequently, the substrate-specific hydrolytic preferences of ACOTs represent a critical regulatory node in TME.

### 2.3 Functional specialization of subcellular compartments

ACOTs are distributed across various subcellular compartments, including the cytoplasm, mitochondria, and peroxisomes, with some exhibiting dual localization. This spatial segregation enables ACOTs to respond precisely to metabolic demands within specific microenvironments. Subcellular compartmentalization is a fundamental feature of acyl-CoA metabolism. Studies have shown that the mitochondrial matrix houses the majority of the cell’s CoA for energy production, while cytoplasmic acyl-CoA is primarily directed toward lipid synthesis pathways (32). Through the strict subcellular localization and substrate specificity, members of the ACOT family precisely regulate the composition and flux of the acyl-CoA pool within distinct organellar microenvironments (23) (**Table 1**).

**Table 1:** Substrate preference and subcellular compartmentalization of human acyl-CoA thioesterases (ACOTs).

Gene ID	Preferred substrate/characteristics	Subcellular localization	Reference
ACOT1	Palmitoyl-CoA, Stearoyl-CoA	Cytosol	(38)
ACOT2	Palmitoyl-CoA, Stearoyl-CoA	Peroxisome, Mitochondrion	(39)
ACOT4	Glutaryl-CoA, long-chain fatty acyl-CoAs (≥C20)	Peroxisome	(40)
ACOT6	phytanoyl-CoA, pristanoyl-CoA (from foods)	Peroxisome, Cytosol	(41)
ACOT7	Palmitoyl-CoA, long-chain fatty acyl-CoAs (≥C20)	Cytosol, Mitochondria	(24)
ACOT8	broad-chain-length acyl-CoAs, CoA esters of bile acids	Peroxisome	(26)
ACOT9	broad-chain-length acyl-CoAs (C2-C20)	Mitochondria	(33)
ACOT11	Palmitoyl-CoA, Myristoyl-CoA	cytosol Mitochondria	(25)
ACOT12	Acetyl-CoA, Propionyl-CoA, Butyryl-CoA	cytosol	(42)
ACOT13	Medium and long-chain fatty acyl-CoA (C12-C18)	Cytosol, nucleus	(43)
THEM4	Palmitoyl-CoA, Oleoyl-CoA	Mitochondria	(44)
THEM5	Medium and long-chain fatty acyl-CoA (C12-C18)	Mitochondria	(45)

Specifically, mitochondria-localized ACOT2 and ACOT9 primarily regulate β-oxidation flux by hydrolyzing long-chain acyl-CoA to prevent mitochondrial lipid overload, while also modulating reactive oxygen species (ROS) production (33, 34). Human peroxisomes primarily contain ACOT4, ACOT8, and ACOT12, which are mainly responsible for the degradation of very-long-chain fatty acids. Among these, ACOT8 has an extremely broad substrate range (C2–C20) and is highly sensitive to CoASH. By sensing CoASH levels, it can optimize fatty acid β-oxidation flux and therefore has become a central hub in peroxisomal lipid metabolism (35).

Most cytoplasmic ACOT members exhibit dual-localization characteristics. For instance, the PTS1 variant (-SKV) present in ACOT1 serves as a “weak” peroxisomal targeting signal, facilitating dual localization to the peroxisomes and either the cytoplasm or mitochondria (36). Additionally, some ACOT members contain a START domain, which dynamically modulates their subcellular localization and enzymatic activity. For example, ACOT11’s START domain can allosterically regulate its enzymatic activity by binding specific long-chain fatty acids (e.g., norcapric acid) or lysophosphatidyl-choline (LPC). This domain also anchors the protein near lipid droplets, enabling precise spatial regulation of its localization and function (37).

In tumor cells, disruption of compartmentalization—such as abnormal expression of the ACOT family—directly reshapes the metabolic flux of acyl-CoA. This can lead to insufficient mitochondrial energy production or aberrant cytoplasmic lipid synthesis, which in turn affects tumor cell proliferation, survival, and signal transduction within the immune microenvironment. These mechanisms will be discussed in detail in the next chapter.

## 3 ACOTs and their derived lipid mediators participate in regulating tumor metabolic reprogramming and microenvironmental remodeling

### 3.1 Expression regulation and functions of ACOTs

ACOTs exhibit significant heterogeneity in their tissue expression, biological functions, and clinical significance in tumors. Most members are upregulated in tumors and have been implicated in tumor progression through multiple regulatory mechanisms, including transcriptional regulation, epigenetic modifications, and non-coding RNAs. Through altering the release profiles of specific lipid mediators, these mechanisms shape the TME and subsequently regulate tumor lipid metabolism, ferroptosis sensitivity, and malignant progression. This suggests that ACOTs may serve as potential markers for precision immunotherapy stratification by regulating the metabolic-immune dialogue within the tumor microenvironment.

#### 3.1.1 Cancer-specific expression of different ACOT

ACOTs exhibit distinct expression patterns across various tumor types and are closely linked to patient prognosis. Genome-wide analyses have identified ACOT1 as a frequently deleted gene in gastric cancer populations, suggesting its potential role as a tumor suppressor in gastric carcinogenesis (46); Similarly, ACOT13 is downregulated in serous ovarian cystic adenocarcinoma and is associated with shorter overall survival, progression-free survival, and disease-specific survival, indicating its function as a tumor suppressor (47).

In contrast, most ACOT family members have been implicated in tumor progression, although their roles appear to be context-dependent and may reflect downstream effects of broader oncogenic programs rather than independent driver functions. For example, genetic analyses have revealed that ACOT2 expression is significantly positively correlated with pancreatic cancer risk (OR 1.35, 95% CI 1.17–1.56), identifying it as a novel pancreatic cancer susceptibility gene. This association is independent of risk SNPs previously identified in GWAS studies, suggesting that ACOT2 may contribute to pancreatic cancer susceptibility by modulating lipid metabolism in normal pancreatic tissue (48). Furthermore, ACOT2 is highly expressed in patients with acute myeloid leukemia (AML) and is strongly associated with poor overall survival (P = 0.003) (49). ACOT4 interacts with B7 homolog 4 (B7H4) in tumor-associated fibroblasts and is linked to poor prognosis in gastric cancer patients (50). ACOT7 has been identified as a novel oncogene and potential therapeutic target in lung adenocarcinoma (51), and silencing ACOT7 has been shown to inhibit the proliferation and metastatic ability of colorectal cancer cells (52). Additionally, ACOT8 expression is dysregulated in multiple cancers, although the direction of change varies by tumor type. It is upregulated in breast cancer and colorectal cancer, where elevated expression correlates with poor prognosis (53, 54). In clear cell renal cell carcinoma, however, ACOT8 is significantly downregulated, yet its decreased expression is also associated with disease progression and unfavorable outcomes (55).

The expression levels of THEM4 are closely associated with the prognosis of patients with various cancers. Although this protein belongs to the ACOT superfamily, research on it has primarily focused on its non-classical function: as an endogenous inhibitor of the AKT kinase, THEM4 suppresses AKT activity by directly binding to it, thereby inhibiting cell proliferation and inducing apoptosis. However, THEM4 exhibits complex and even contradictory functional characteristics across different tumor types. Studies have shown that THEM4 is a potential diagnostic biomarker for poor recurrence and disease-free survival in patients with triple-negative breast cancer, and it promotes metastasis in triple-negative breast cancer through AKT-activation-dependent pathways (56). Conversely, CAMK2A inhibits the progression of pancreatic ductal adenocarcinoma (PDAC) by impairing mitochondrial function (reducing membrane potential), ATP production, and ROS levels, and by promoting the release of THEM4 from mitochondria (57). These contradictions suggest that the function of THEM4 is likely context-dependent, with its roles varying across cancer types and microenvironmental settings. This highlights the need for further investigation into the precise conditions under which THEM4 exerts pro- or anti-tumorigenic effects.

#### 3.1.2 Upstream regulatory mechanisms of ACOTs

The expression of ACOTs is intricately regulated by various transcription factors and non-coding RNAs. The nuclear receptor PPARα directly governs the transcription of multiple ACOTs. A landmark study demonstrated that PPARα binds to the DR1 element in the Acot1 promoter region. Treatment with PPARα agonists increased Acot1 mRNA levels by up to 90-fold in the liver, whereas this induction was completely absent in PPARα-knockout mice (58). Intriguingly, free fatty acids produced by ACOT enzymatic hydrolysis can serve as endogenous ligands for PPARα, activating its transcriptional activity and establishing a positive feedback loop between ACOT and PPARα (59). This feedback loop allows ACOTs to sense intracellular fatty acid fluctuations and convert metabolic signals into transcriptional responses, dynamically maintaining lipid metabolic homeostasis. However, whether this feedback loop operates similarly in the tumor microenvironment remains an open question. The physiological rheostat function of the ACOT-PPARα loop may be overridden during malignant transformation, potentially due to aberrant oncogenic signaling, metabolic rewiring, or tissue-specific factors that disrupt the normal coupling between fatty acid availability and ACOT transcription.

In addition, other transcription factors also regulate the expression of ACOTs in tumors. For instance, the transcription factor KLF13 directly binds to the ACOT7 promoter region, activating its transcription (60). Similarly, in diffuse large B-cell lymphoma (DLBCL), SREBF2 transcriptionally activates ACOT7 expression. The upregulated ACOT7 promotes tumor cell proliferation and survival by reshaping lipid metabolism (61). In non-small cell lung cancer (NSCLC), ARNTL2 directly activates ACOT7 expression, with resultant upregulation promoting tumor proliferation via dual mechanisms of inhibiting apoptosis and ferroptosis (62).

The ACOTs also subject to post-transcriptional regulation by non-coding RNAs. For example, in breast cancer cells (MCF7), ACOT7 mRNA decay is triggered by the microRNA miR-9 in a WIG1-dependent manner via classic recruitment of AGO2 (63); In gliomas, glioma stem cell-like cell-derived exosomes carry miR-155-5p, which binds specifically to the 3’UTR of ACOT12, inhibiting its mRNA and protein expression. Downregulation of ACOT12 results in elevated intracellular acetyl-CoA levels, histone hyperacetylation, and subsequent stromal transformation in tumor cells, ultimately promoting glioma invasion and malignant progression (64). Similarly, in colorectal cancer, exosomes derived from M2-type tumor-associated macrophages carry miR-183-5p, which specifically inhibits THEM4 expression. This inhibition results in sustained activation of the PI3K/AKT and NF-κB signaling pathways, thereby promoting tumor cell proliferation, migration, invasion, and epithelial-mesenchymal transition (65).

**Figure 2:**
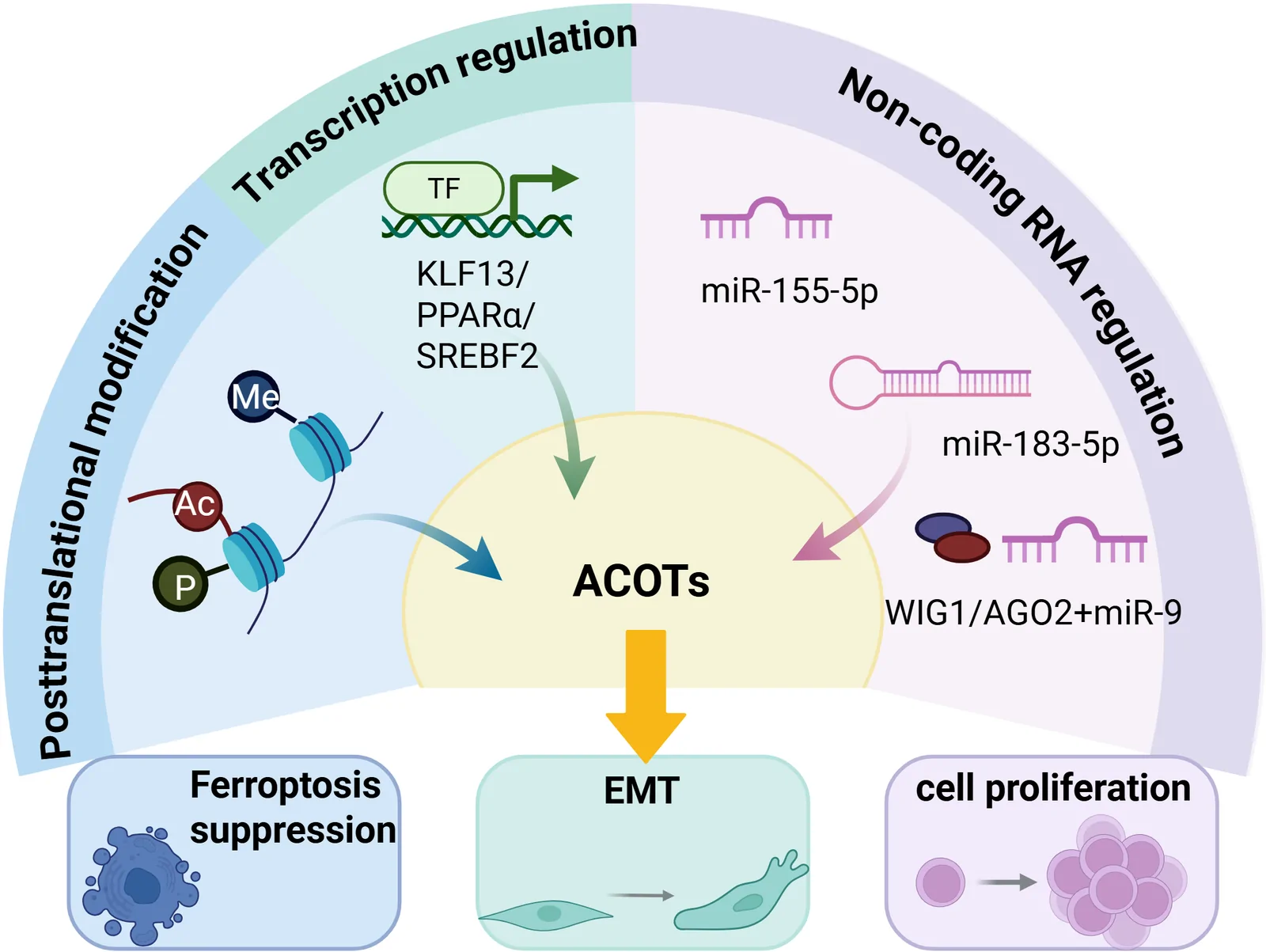
Schematic overview of the regulatory landscape of ACOT family expression in cancer. ACOT members are regulated at three interconnected levels: transcriptional regulation by transcription factors, post-transcriptional regulation by non-coding RNAs, and post-translational regulation via protein phosphorylation, acetylation and methylation. These layers collectively shape ACOT expression and function, impacting ferroptosis, EMT, and tumor cell survival and proliferation. Created in BioRender. Ren. (2026) https://BioRender.com/o5dqkto.

ACOTs are also regulated by post-translational modifications (PTMs). In ovarian cancer, NAT10-mediated acetylation of ACOT7 enhances its stability and translation efficiency. This regulatory axis suppresses ferroptosis by remodeling fatty acid metabolism, thereby driving malignant tumor progression (66). In gastric cancer, the long non-coding RNA (lncRNA) NMRAL2P indirectly induces methylation in the ACOT7 promoter region by binding to DNMT3b, thereby suppressing ACOT7 expression. This methylation-regulated axis also contributes to the malignant progression of gastric cancer (67). On the other hand, ACOT4 is primarily regulated by phosphorylation, which affects its protein stability. Mechanistic studies have shown that AKT phosphorylates ACOT4 at the S392 site. This modification reduces the binding of ACOT4 to HSPA1A, thereby prolonging the half-life of the ACOT4 protein and leading to its accumulation in tumor cells. Elevated ACOT4 levels support the metabolic demands of tumor cells by producing excessive amounts of coenzyme A (CoA), thereby promoting the formation and proliferation of pancreatic tumors (68) (**Figure 2**).

#### 3.1.3 ACOTs regulate lipid metabolism and the tumor mircroenvironment

The ACOTs influence tumor cell fate and the TME by regulating lipid metabolism. Ferroptosis, an iron-dependent form of programmed cell death, is closely linked to the peroxidation of intracellular polyunsaturated fatty acid (PUFA) phospholipids. ACOTs regulate membrane lipid composition and lipid peroxidation levels by hydrolyzing specific acyl-CoA species, thereby influencing ferroptosis susceptibility. For example, ACOT7 hydrolyzes polyunsaturated acyl-CoA species, such as arachidonyl-CoA, reducing the pool of PUFA substrates available for membrane phospholipid synthesis. This limits the availability of lipid peroxidation substrates, enabling metabolic reprogramming of ferroptosis sensitivity under the upstream regulation of transcription factor activation (62).

ACOTs also play a key role in the regulation of TME. For instance, in patients who respond to anti-PD-L1 therapy, ACOT2 expression levels are significantly higher than in non-responders, suggesting that it may influence the characteristics of the tumor immune microenvironment by regulating the release of lipid mediators, thereby demonstrating potential as a biomarker for predicting immunotherapy response (69). Besides, Inflammatory signals within the tumor microenvironment upregulate ACOT7, which remodels the composition of phospholipids containing long-chain (≥C20) unsaturated acyl chains in macrophages. This remodeling regulates cell membrane lipid homeostasis and influences immune responses (70). ACOT7 has also been identified as a mitochondria-associated prognostic biomarker that contributes to the reprogramming of primary bile acid anabolism, which subsequently modulates immune cell infiltration and function in the tumor microenvironment (71). Similarly, in invasive ductal carcinoma of the breast, ACOT7 regulates immune cell infiltration and function by reshaping lipid metabolic homeostasis. This activity promotes the formation of an immunosuppressive tumor microenvironment and facilitates immune evasion (72). In hepatocellular carcinoma, ACOT12-dependent accumulation of acetyl-CoA activates TWIST2 gene expression via epigenetic pathways, enhancing cancer cell migratory capacity. Meanwhile, tumor-associated macrophages secrete acetic acid through the lipid peroxidation-ALDH2 pathway, supplying precursors for acetyl-CoA synthesis in cancer cells and forming a metabolic feedback loop between tumor cells and microenvironmental cells (73).

Regarding the regulation of antigen presentation, it is reported that ACOT expression in microsatellite-stable colorectal cancer was negatively correlated with overall patient survival. Low ACOT expression was associated with increased MHC-I molecule expression and improved response to immunotherapy. Mechanistic studies revealed that THEM4 interacts with the mitotic regulator REV7, disrupting its negative regulation of CDK1. Under conditions of THEM4 knockdown or REV7 overexpression, tumor cells exhibited G2 phase arrest, MHC-I expression was significantly upregulated, and CD8^+^ T-cell infiltration and tumor-killing activity were enhanced upon IFN-γ stimulation, thereby boosting immunogenicity. This finding represents a novel mechanism by which ACOT family members regulate MHC-I molecule expression and T-cell immune responses, underscoring their potential as immune regulatory factors (74).

### 3.2 Interaction of ACOT-derived lipid mediators with the TME

As previously mentioned, ACOTs serve as a key driver of metabolic reprogramming in the tumor immune microenvironment by regulating the dynamic balance of the acyl-CoA pool. On the substrate side, long-chain and short-chain acyl-CoA substrates processed by ACOT hydrolysis directly influence the fate of tumor and immune cells through protein acylation modifications, membrane phospholipid composition, and metabolic signaling pathways. On the product side, the regional accumulation of monounsaturated fatty acids (MUFAs) creates an immunosuppressive microenvironment, driving M2-like macrophage polarization and inhibiting CD8^+^ T-cell effector functions (31); the release of polyunsaturated fatty acids (PUFAs) acts as a critical metabolic switch, converting “cold” tumors into “hot” tumors by fostering an inflammatory microenvironment (75). Specifically, PUFA-derived arachidonic acid specifically recruits and activates innate immune cells, initiating localized inflammatory responses. Additionally, the incorporation of PUFAs into membrane phospholipids induces immunogenic ferroptosis in tumor cells (76). In contrast, the accumulation of saturated fatty acids (SFAs) contributes to the release of pro-inflammatory factors while simultaneously triggering immunosuppressive signals, such as activating the NLRP3 inflammasome via the TLR4-NF-κB axis (77).

These lipid signals arising from ACOT enzymatic activity synergize with metabolic stresses—including hypoxia and acidic conditions—to collectively establish an immunosuppressive microenvironment conducive to tumor progression. Furthermore, these lipid mediators can exert systemic effects by influencing draining lymph nodes and distant metastatic sites either via exosomes or in their free form (78), thereby constructing a comprehensive metabolic-immune regulatory network.

#### 3.2.1 Product side: free fatty acids

##### 3.2.1.1 SFAs

SFAs, particularly palmitic acid (C16:0), act as critical metabolic signaling molecules in the tumor microenvironment. Through mechanisms such as pattern recognition receptor activation, induction of endoplasmic reticulum stress, and post-translational protein modification, SFAs reshape innate and adaptive immune responses (79). Multiple members of ACOTs (ACOT1, ACOT2, ACOT4) exhibit high hydrolytic activity toward palmitoyl-CoA, regulating its local availability by converting saturated acyl-CoA into free palmitic acid and coenzyme A. Studies have shown that palmitic acid promotes the palmitoylation of B7H3 via the enzyme ZDHHC24 (palmitoyltransferase), which limits its autophagic degradation and stabilizes the B7H3 protein. This stabilization inhibits the antitumor activity of CD8^+^ T cells (80). In hepatocellular carcinoma, palmitic acid-induced lipid accumulation upregulates PD-L1 expression and promotes the polarization of macrophages toward an M2-like immunosuppressive phenotype. Simultaneously, it activates hepatic stellate cells to secrete TGF-β, thereby fostering an immunosuppressive microenvironment (81). Furthermore, excessive free palmitic acid produced by tumor cells has been shown to enter CD8^+^ T cells, induce STAT3 palmitoylation, and drive terminal exhaustion of CD8^+^ T cells. This mechanism contributes to resistance against anti-PD-1 therapy (82).

##### 3.2.1.2 MUFAs

Unlike SFAs, MUFAs, such as oleic acid (C18:1), exhibit distinct and complex regulatory roles in tumor immunity. Studies have revealed opposing effects of oleic acid and palmitic acid on γδ T cells. While palmitic acid induces excessive IFN-γ secretion in γδ T cells, triggering pyroptosis and impairing their antitumor activity, oleic acid reverses these effects by reducing IFN-γ secretion and alleviating pyroptosis. This highlights the critical influence of fatty acid composition on immune cell function (83). Clinical studies have demonstrated that changes in MUFA levels in peritumoral tissues are closely correlated with CD163^+^ TAM (tumor-associated macrophage) infiltration. Particularly, one cycle of neoadjuvant chemotherapy resulted in a percentage change in MUFA levels that positively correlated with the expression of CD163, indicating that MUFA remodeling contributes to macrophage polarization toward an M2-like immunosuppressive phenotype (84). Members of ACOTs—including ACOT1 and ACOT2—possess hydrolytic activity toward monounsaturated acyl-CoA substrates. These enzymes produce free oleic acid and coenzyme A by hydrolyzing oleoyl-CoA, directly modulating MUFA levels and signaling intensity within the tumor microenvironment (49).

##### 3.2.1.3 SUFAs

**Figure 3:**
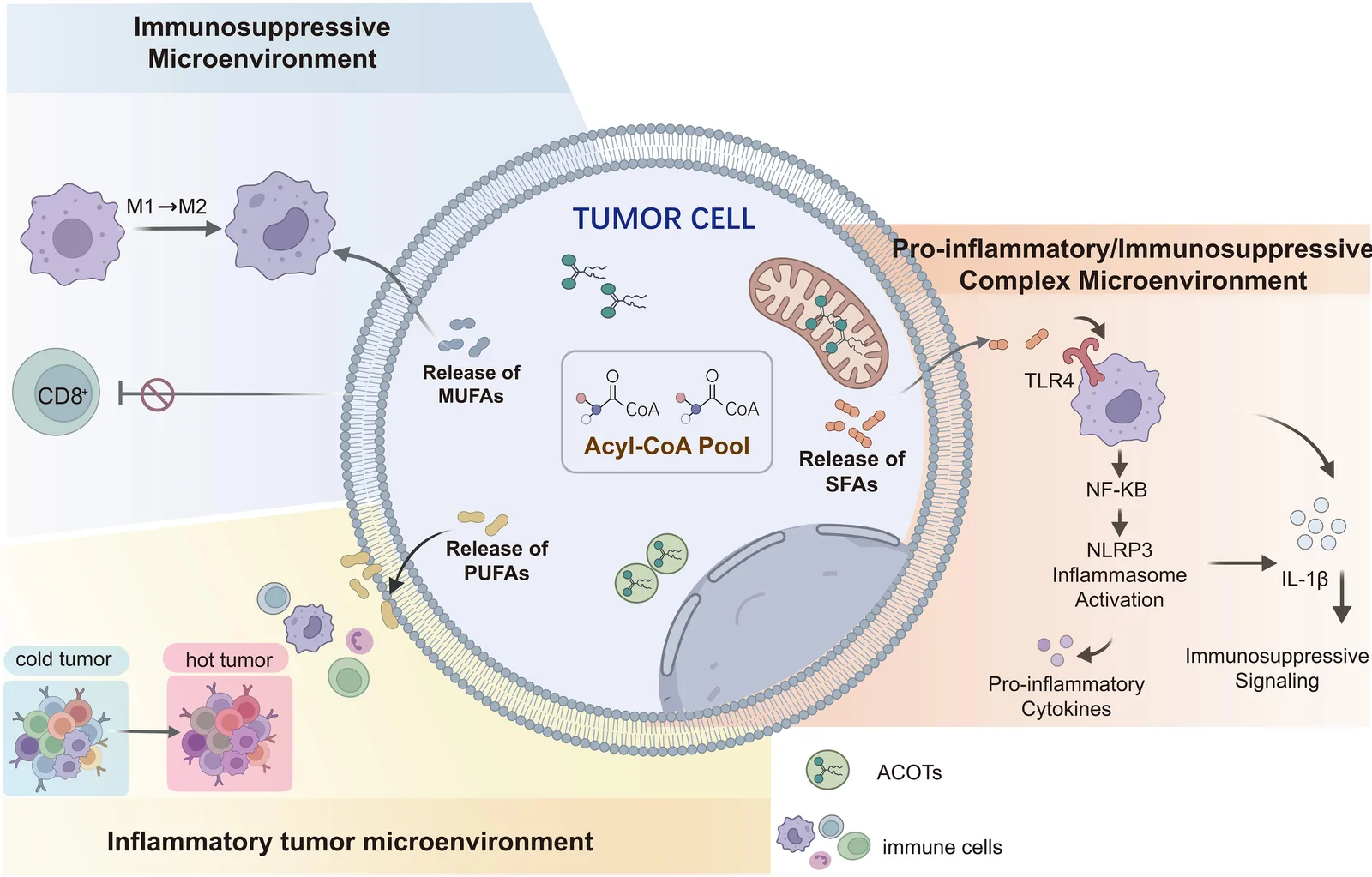
ACOTs hydrolyze acyl-CoA to regulate the balance of free fatty acids (SFAs, MUFAs, and PUFAs) within the tumor microenvironment. SFAs activate the TLR4/NF-κB/NLRP3 pathway to promote immunosuppressive signaling, while PUFAs contribute to an inflammatory microenvironment that facilitates the conversion of “cold” tumors to “hot” tumors, thereby enhancing anti-tumor immunity. Created in BioRender. Ren. (2026) https://BioRender.com/9owyw62.

ACOTs also display hydrolytic activity toward polyunsaturated acyl-CoA. Among them, ACOT8 demonstrates the highest hydrolytic activity for polyunsaturated fatty acids (PUFAs), efficiently catalyzing the hydrolysis of polyunsaturated acyl-CoA substrates, such as linoleoyl-CoA (C18:2) and arachidonyl-CoA (C20:4). Additionally, other ACOTs localized to distinct cellular compartments also exhibit significant hydrolytic activity toward unsaturated acyl-CoA species. For instance, ACOT7 exhibits a high hydrolytic preference for arachidonyl-CoA and has been identified as a poor prognostic factor in various cancer types, suggesting its role in tumor progression by modulating arachidonic acid levels (85). In the context of tumor immune regulation, PUFAs exhibit a complex dual regulatory profile within the tumor immune microenvironment (86). Arachidonic acid (AA) is one of the most prominent PUFAs. Enzymes involved in AA metabolism, such as cyclooxygenase (COX), catalyze the production of key precursors of pro-inflammatory lipid mediators, including prostaglandin E2 (PGE2) and leukotrienes. Among these, PGE2 stands out as a potent immunosuppressive mediator. It inhibits dendritic cell maturation, promotes M2-type tumor-associated macrophage polarization, and induces CD8^+^ T-cell exhaustion via the EP2/EP4 receptor signaling pathway, contributing to the establishment of an immunosuppressive microenvironment (87). However, the regulatory effects of arachidonic acid metabolism are not uniformly immunosuppressive. For instance, in non-small cell lung cancer, 5-lipoxygenase (ALOX5) expressed in tumor cells metabolizes arachidonic acid to generate leukotriene B4 (LTB4). This lipid mediator promotes the recruitment of CD8^+^ T cells to the tumor site, thereby inhibiting tumor growth and exhibiting synergistic effects with anti-PD-1 therapy (88) (**Figure 3**).

#### 3.2.2 Substrate side: Acyl-CoA

##### 3.2.2.1 Short-chain Acyl-CoA

Short-chain acyl-CoA (SCACoA), primarily consisting of acetyl-CoA and propionyl-CoA, plays essential roles in cellular metabolism. Acetyl-CoA serves as a central metabolic hub, integrating the tricarboxylic acid cycle, fatty acid synthesis, and epigenetic modifications (89). In the TME, acetyl-CoA metabolism significantly influences the epigenetic states and functional polarization of immune cells. For instance, acetic acid taken up by tumor cells is converted into acetyl-CoA, elevating intracellular acetyl-CoA levels. This elevation promotes lysine 148 acetylation of c-Myc, stabilizing c-Myc and transcriptionally activating PD-L1 expression, ultimately inhibiting CD8^+^ T-cell infiltration and facilitating immune evasion (90). Additionally, elevated acetyl-CoA levels in tumor-infiltrating CD8^+^ T cells lead to lipid metabolism dysfunction and mitochondrial impairment, subsequently weakening their antitumor activity (91).

propionyl-CoA metabolism primarily impacts immune cell function through two mechanisms. First, propionyl-CoA serves as an acyl donor for histone propionylation, altering epigenetic regulation. For example, in regulatory T cells (Tregs), heightened acyl-CoA metabolic flux enhances the expression of Treg-associated genes via histone propionylation, thereby maintaining Treg functionality and immune tolerance (92). Second, abnormalities in acyl-CoA metabolism can modify immune cell states. For example, in pancreatic cancer, the loss of the propionyl-CoA carboxylase B subunit (PCCB) leads to the accumulation of propionyl-CoA, which in turn results in the inactivation of CD8^+^ T cells infiltrating the tumor. Although these T cells continue to infiltrate the tumor tissue, their antitumor activity is significantly impaired (93).

The ACOT family directly regulates intracellular steady-state levels and metabolic flux of short-chain acyl-CoA molecules by hydrolyzing them, thereby impacting downstream immune signaling. Among ACOT family members, ACOT12 exhibits the highest hydrolytic specificity for short-chain acyl-CoA, efficiently catalyzing the hydrolysis of acetyl-CoA and propionyl-CoA into free acetic acid, propionic acid, and coenzyme A (94). ACOT12 inhibits the metastasis of intrahepatic cholangiocarcinoma cells by lowering acetyl-CoA levels and histone acetylation (95). The underlying mechanism involves ACOT12 hydrolyzing acetyl-CoA, thereby reducing intracellular acetyl-CoA levels, which in turn decreases histone acetylation. This reduction lifts epigenetic repression of transcription factors associated with epithelial-to-mesenchymal transition (EMT), inducing EMT and promoting tumor cell invasion and metastasis (96). These findings demonstrate that ACOT12 exerts critical effects on tumor biology by modulating short-chain acyl-CoA metabolism.

##### 3.2.2.2 Long-chain acyl-CoA

The ACOT family exhibits hydrolytic activity toward long-chain acyl-CoA (LC-CoA) molecules, and numerous studies have confirmed their involvement in signal regulation within the tumor immune microenvironment. Among these, palmitoyl-CoA, oleoyl-CoA, and arachidonyl-CoA play particularly critical roles.

**Figure 4:**
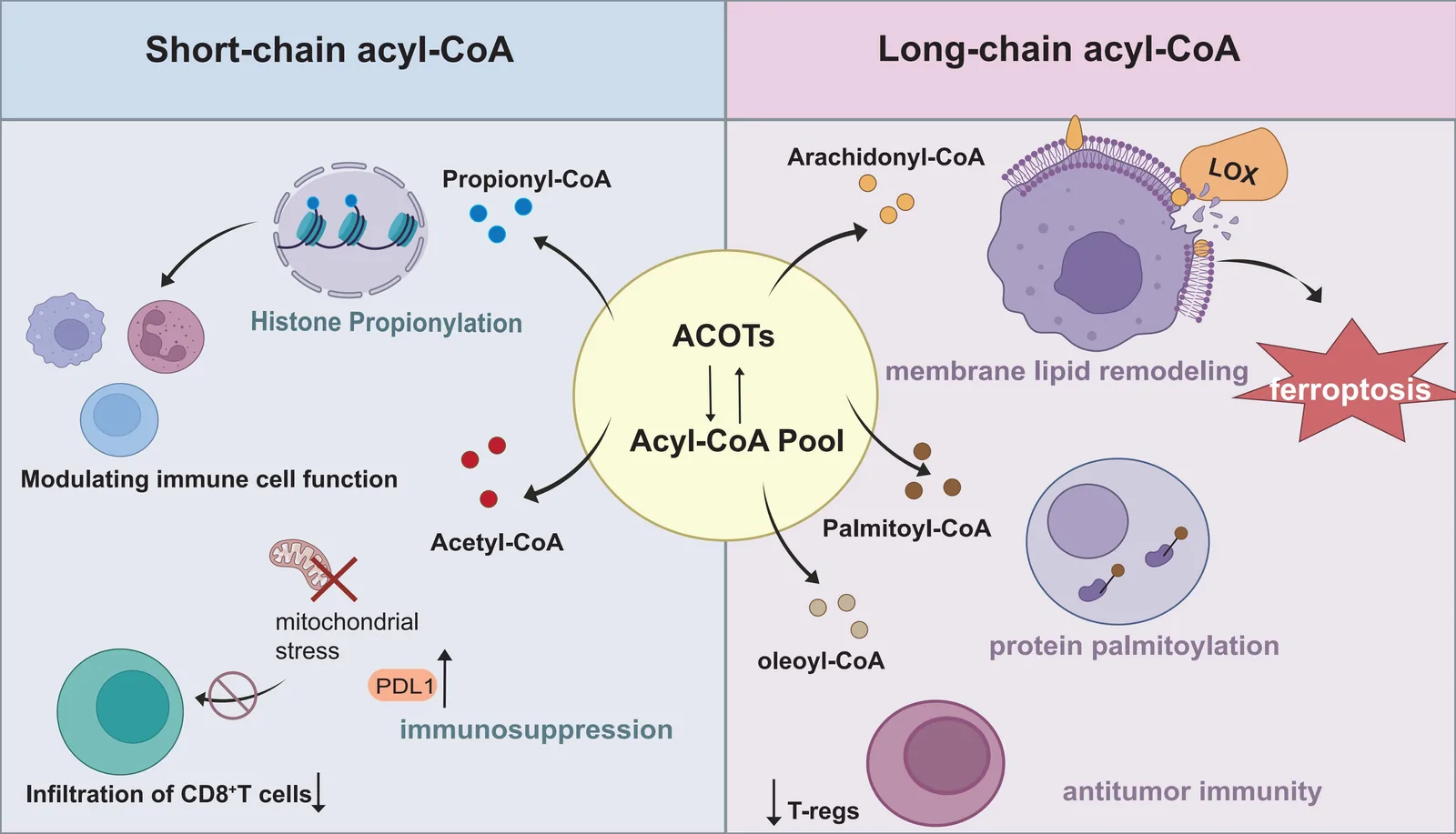
ACOTs can hydrolyze acyl-CoA molecules of varying chain lengths. Different types of acyl-CoA regulate tumor progression and the TME through their respective downstream signaling pathways. Specifically, acetyl-CoA modulates immune cell function via histone acetylation; propionyl-CoA regulates histone acetylation, thereby maintaining Treg cell function and an immunosuppressive microenvironment. Palmitoyl-CoA primarily regulates protein palmitoylation to promote tumor progression; arachidonyl-CoA primarily influences tumor lipid peroxidation and ferroptosis through membrane lipid remodeling, and oleoyl-CoA promotes CD8^+^ T cell antitumor responses, while its decline is associated with immunosuppression. Created in BioRender. Ren. (2026) https://BioRender.com/muvesk3.

Palmitoyl-CoA serves as both a central component of saturated fatty acid metabolism and a regulator of immune processes by acting as an acyl donor for protein palmitoylation (97). Through ZDHHC family palmitoyltransferases, palmitoyl-CoA modulates the stability and membrane localization of immune-related proteins such as PD-L1 and STING, thereby influencing antitumor immune responses (98). Oleoyl-CoA likewise plays a significant regulatory role in tumor immunity. Studies have demonstrated that fatty acids released by cancer-associated fibroblasts (CAFs) within the tumor microenvironment increase oleoyl-CoA and oleic acid levels in CD4^+^ T cells. This metabolic shift promotes Th1 cell differentiation and enhances the antitumor activity of CD8^+^ T cells. Conversely, decreased levels of oleoyl-CoA are strongly associated with the upregulation of Treg cell markers and the establishment of an immunosuppressive microenvironment (99). Unlike the immune-enhancing or inhibitory roles of palmitoyl-CoA and oleoyl-CoA, arachidonyl-CoA primarily influences cell fate through its involvement in membrane phospholipid remodeling. As a key precursor for phospholipid synthesis, arachidonyl-CoA facilitates the incorporation of arachidonic acid (AA) into polyunsaturated fatty acid chains of membrane phospholipids (100). During ferroptosis, arachidonyl-CoA-rich membrane phospholipids are oxidized by lipoxygenases (LOX), triggering a phospholipid peroxidation cascade that ultimately compromises cell membrane integrity and induces ferroptosis (101). Ferroptotic tumor cells release damage-associated molecular patterns (DAMPs), which activate dendritic cells and promote CD8^+^ T cell-mediated antitumor immune responses (102) (**Figure 4**).

### 3.3 ACOTs and other metabolic enzymes jointly regulate Acyl-CoA metabolism and immune signaling

The regulation of lipid mediator profiles by ACOTs is not an isolated process but part of a complex interactive network involving upstream acyl-CoA synthetases, downstream lipid metabolic enzymes and transporters, and other lipid mediators derived from lipid metabolic pathways (23). Together, these interactions orchestrate lipid metabolic reprogramming, regulate downstream signaling, and contribute vitally to the metabolic regulation of the tumor immune microenvironment.

Alterations in the lipid derivative profile due to changes in ACOT activity can create feedback loops that modify the activity and expression of other lipid metabolic enzymes, establishing a dynamic balance within the metabolic network. For instance, in hepatocellular carcinoma, ACOT12 suppresses tumor growth by limiting glycerophospholipid biosynthesis via acetyl-CoA hydrolysis. Loss of ACOT12 function causes an abnormal accumulation of glycerophospholipids, including lysophosphatidic acid (LPA), in the liver. Elevated LPA levels inhibit YAP phosphorylation in the Hippo signaling pathway by activating the LPA receptor and the Gα12/13 signaling cascade, resulting in YAP nuclear translocation and increased transcriptional activity. These changes accelerate the development and progression of hepatocellular carcinoma (103). Furthermore, LPA, a bioactive lipid, promotes polarization of tumor-associated macrophages toward an M2-like phenotype and suppresses CD8^+^ T-cell infiltration, suggesting that ACOT12 deficiency may drive immunosuppressive microenvironment formation through LPA accumulation (104). In addition, ACOT1 catalyzes the hydrolysis of acyl-CoA to produce free fatty acids via its thioesterase activity. These free fatty acids act as endogenous ligands that activate PPARα transcriptional activity, enhancing hepatic fatty acid oxidation and reducing oxidative stress and inflammation caused by excessive oxidation (105). This mechanism has also been validated in non-tumor diseases. For instance, in cardiomyocytes, ACOT1 modifies the free fatty acid profile by hydrolyzing long-chain acyl-CoA, reducing PUFA levels and decreasing lipid peroxidation substrates. This activity has been shown to prevent doxorubicin-induced ferroptosis and protect cardiac function (106).

**Figure 5:**
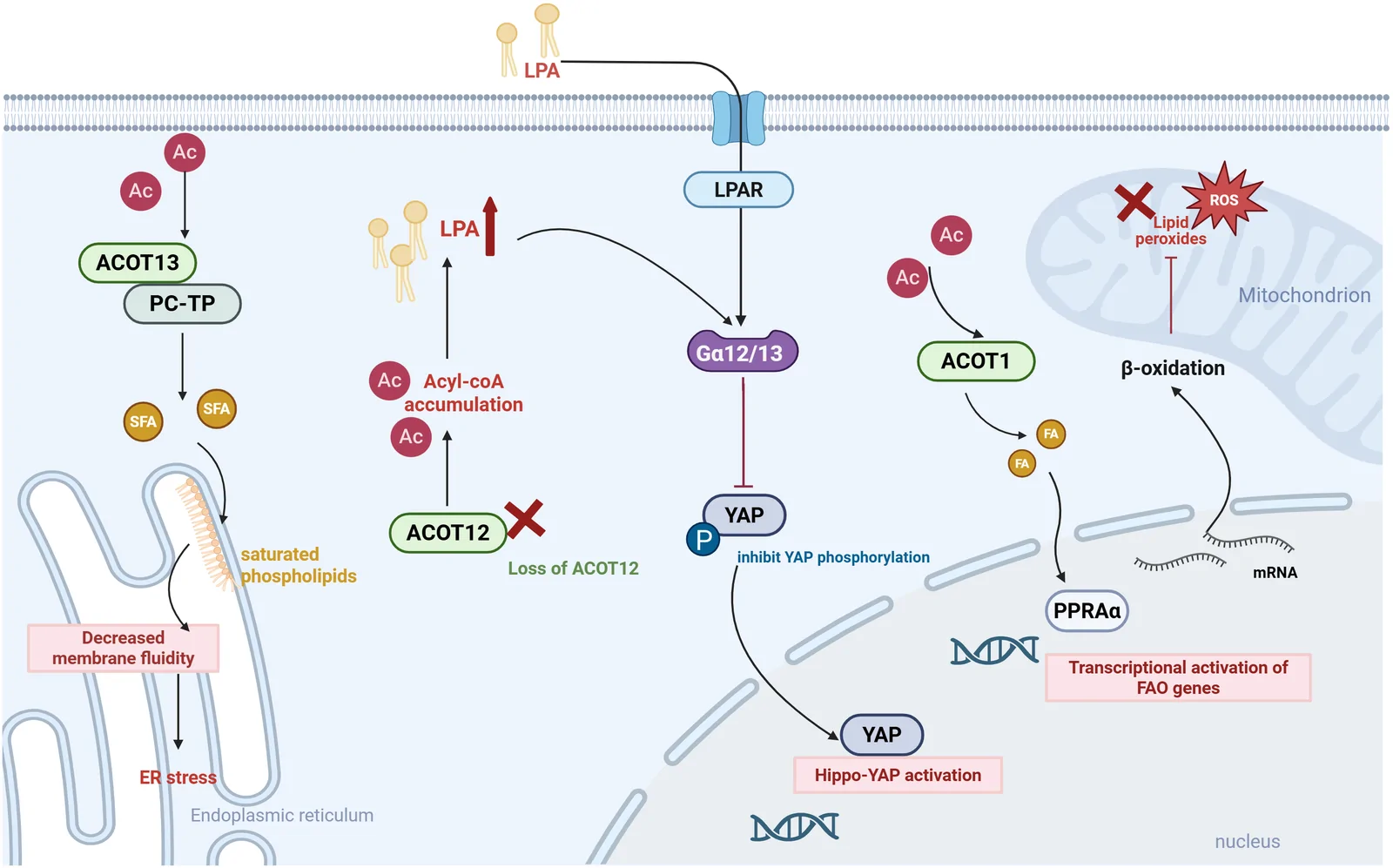
ACOTs integrate with metabolic enzymes to regulate acyl-CoA metabolism and immune signaling. ACOTs function within a complex interactive network involving upstream acyl-CoA synthetases and downstream lipid metabolic enzymes. ACOT12-mediated acetyl-CoA hydrolysis limits glycerophospholipid biosynthesis and LPA accumulation, thereby suppressing YAP signaling. ACOT1-derived free fatty acids activate PPARα to regulate fatty acid oxidation. ACOT13 forms a complex with PC-TP to modulate ER stress. Created in BioRender. Ren. (2026) https://BioRender.com/qcdlt58.

Members of ACOTs also cooperate with other lipid metabolic enzymes to regulate key metabolic processes. For example, thioesterase superfamily member 2 (THEM2/ACOT13) forms a functional complex with phosphatidylcholine transfer protein (PC-TP). Under conditions of chronic overnutrition or acute endoplasmic reticulum (ER) stress, this complex redirects saturated fatty acids from the β-oxidation pathway toward the glycerophospholipid biosynthesis pathway, facilitating the production of saturated phospholipids and reducing ER membrane fluidity. This mechanism activates the ER stress response by enhancing transposon-mediated ER calcium efflux (43). Activation of the ER stress-associated IRE1α-XBP1 pathway has been shown to promote M2-like polarization of tumor-associated macrophages, suggesting that the ACOT13-PC-TP complex mediates tumor microenvironment dynamics via metabolic-immune crosstalk (107). Synergistic regulatory effects between ACOTs and other metabolic enzymes have also been identified in non-tumor diseases. For instance, in diabetic cardiomyopathy, increased LPCAT3 expression and reduced ACOT1 expression result in a metabolic imbalance that promotes the accumulation of lipid peroxides in cardiomyocytes. This accumulation enhances the incorporation of PUFAs into phospholipid membranes, leading to ferroptosis and worsened cardiac dysfunction (108) (**Figure 5**).

In summary, this multi-enzyme interactive network positions the ACOT family as a central node that integrates fatty acid uptake, storage, oxidation, and signaling output. This regulatory capacity suggests that the ACOT family may contribute to shaping the metabolic characteristics and functional phenotypes of the tumor immune microenvironment, although whether they act as primary drivers or as components of broader oncogenic programs remains to be determined.

## 4 Intervention strategies targeting Acyl-CoA immunometabolism and the translational prospects of ACOTs

As highlighted earlier, ACOTs function as “hydrolysis nodes” within the acyl-CoA metabolic network, forming a finely regulated metabolic loop alongside acyl-CoA synthetases, fatty acid synthases, and fatty acid β-oxidation nodes. The functional integrity of this metabolic network relies on the coordination of all nodes; an imbalance in any single node can trigger metabolic reprogramming, driving tumor progression and remodeling the tumor microenvironment.

From a translational medicine perspective, targeting synthetic nodes within the acyl-CoA metabolic network represents an upstream regulation strategy, as it directly affects the acyl-CoA pool maintained by ACOTs. Conversely, targeting fatty acid β-oxidation can block downstream signaling mediated by ACOT-derived lipid mediators. While no drugs directly targeting ACOTs have yet entered clinical trials, progress in the development and testing of drugs targeting upstream and downstream nodes, such as fatty acid synthase (FASN) and acyl-CoA synthetase short-chain family member 2 (ACSS2), highlights the feasibility of the acyl-CoA metabolic network as an antitumor target (109). Such progress also suggests potential for combining these drugs with immunosuppressive therapies. Furthermore, this work provides critical proof of concept and establishes a foundational framework for the future development of ACOT-targeting therapeutic.

This section will review the clinical and preclinical advancements of representative drugs targeting metabolic nodes within the acyl-CoA network. Based on these findings, it will discuss potential strategies and challenges for leveraging ACOTs as novel intervention targets in cancer therapy.

### 4.1 Clinical progress of drugs targeting Acyl-CoA metabolizing enzymes

Recent progress in targeting lipid metabolism is supported by evidence showing that tumors are highly dependent on *de novo* lipogenesis. This dependency is not restricted to a single cancer type. Cancers driven by MYC, RAS, or BCR-ABL all converge on lipid synthesis as a common metabolic vulnerability (110). Inhibition of this pathway selectively eliminates tumor cells while sparing normal cells. For instance, in IDH1-mutant acute myeloid leukemia, the dependency is further refined: these tumors exhibit synthetic lethality upon ACC1 inhibition, a key enzyme in *de novo* lipogenesis (111). Collectively, these observations establish lipogenesis as a broadly shared target across diverse oncogenic contexts. The following sections review the clinical and preclinical progress of representative agents targeting Acyl-CoA metabolism.

#### 4.1.1 FASN inhibitors: TVB-2640

FASN inhibitors represent the most advanced class of acyl-CoA-targeting drugs. The representative drug TVB-2640 (Denifanstat) reversibly inhibits FASN, blocking the conversion of acetyl-CoA and malonyl-CoA into palmitoyl-CoA, thereby directly reducing long-chain saturated acyl-CoA levels (112). In hepatocellular carcinoma, genetic or pharmacological inhibition of FASN enhances MHC-I expression in tumor cells, promotes antigen presentation, and stimulates the cytotoxic activity of antigen-specific CD8^+^ T cells (113).

Several clinical trials are investigating FASN inhibitors in combination with immunotherapy or chemotherapy. For HER2-positive breast cancer, a Phase II study combining FASN inhibitors with trastuzumab plus paclitaxel is ongoing (NCT03179904). Meanwhile, in recurrent high-grade astrocytoma, a Phase II trial combining TVB-2640 with bevacizumab demonstrated a 31.4% 6-month progression-free survival (PFS6) in the combination group—a significant improvement over historical data for bevacizumab monotherapy (16%) (P = 0.008)—with a favorable safety profile (114). Based on these findings, China has initiated a Phase III clinical trial (NCT05118776) evaluating the combination of TVB-2640 (ASC40) and bevacizumab for recurrent glioblastoma; however, results have not yet been published.

#### 4.1.2 ACSS2 inhibitors: MTB-9655

MTB-9655 is the first acetyl-CoA synthetase-targeted drug to enter clinical trials. This selective ACSS2 inhibitor blocks acetate-dependent acetyl-CoA synthesis, thereby reducing intracellular acetyl-CoA levels (115). Currently, its Phase I study (NCT04990739) has completed dose escalation in patients with advanced solid tumors. A 2022 ASCO report highlighted that no dose-limiting toxicities were observed in 10 patients, with nausea (29%) being the most commonly reported adverse event. Drug exposure reached the anticipated level of biological activity (116).

#### 4.1.3 Other enzyme inhibitors

In addition to FASN and ACSS2 inhibitors, small-molecule inhibitors targeting several other key enzymes in the acyl-CoA metabolic network have demonstrated potential in preclinical studies for combination with immunotherapy. ACC inhibitors ND-646 and ND-654 block the conversion of acetyl-CoA to malonyl-CoA, significantly inhibiting tumor growth. These inhibitors induce endoplasmic reticulum stress and oxidative damage, triggering tumor cell apoptosis and increasing tumor immunogenicity (117). ACLY inhibitors such as BMS-303141 impair acetyl-CoA production, affecting histone acetylation. In T-cell lymphomas (T-NHLs) with inactivating PD-L1 mutations, ACLY inhibition disrupts abnormal AP-1 signaling in PD-1-deficient T-NHLs and ameliorates immune dysfunction (118). CPT1 inhibitors, such as Etomoxir, prevent long-chain acyl-CoA from entering mitochondria for β-oxidation (119). Studies have shown that Etomoxir reduces the immunosuppressive activity of myeloid-derived suppressor cells (MDSCs) and promotes ferroptosis in MDSCs, reversing their suppression of CD8^+^ T cells (120). Etomoxir has also been shown to reduce the proportion of PD-1-high-expressing CD4^+^ T cells while enhancing IFNγ, perforin, and granzyme B expression in CD8^+^ T cells (121).

These preclinical studies suggest that inhibitors targeting key enzymes in the acyl-CoA metabolic network may provide novel strategies for combination therapy by reshaping tumor metabolism and modulating the immune microenvironment (**Table 2**).

**Table 2:** Preclinical and clinical inhibitors targeting key enzymes in acyl-CoA metabolism.

Target	Agent	Mechanism	Phase	Cancer type	ClinicalTrials.gov identifier
FASN	TVB-2640 (Denifanstat)	Inhibits palmitoyl-CoA synthesis	Phase II Phase II Phase II Phase III	HER2+ BC, NSCLC rHGA rGBM	NCT03179904 NCT03808558 NCT03032484 NCT05118776
	GSK2194069	Inhibits palmitoyl-CoA synthesis	Preclinical	LC	/
	IPI-9119	Inhibits palmitoyl-CoA synthesis	Preclinical	Prostate cancer	/
ACSS2	MTB-9655	Inhibits acetyl-CoA synthesis	Phase I	AST	NCT04990739
ACC	ND-646	Inhibits malonyl-CoA synthesis	Preclinical	NSCLC	/
	ND-654	Inhibits malonyl-CoA synthesis	Preclinical	HCC	/
ACLY	BMS-303141	Inhibits acetyl-CoA synthesis	Preclinical	AST	/
CPT1	Etomoxir	Competes acyl-CoAs for binding to CPT1	Preclinical	Prostate cancer	/

### 4.2 Prospects and challenges of ACOTs as potential drug targets

#### 4.2.1 Functional validation of ACOTs as therapeutic targets

Several ACOT family members promote tumor progression and treatment resistance, making them potential therapeutic targets. In hepatocellular carcinoma, ACOT2 has been identified as a target of the small-molecule compound MI202. Its downregulation correlates with reduced MYCN expression and tumor cell apoptosis, suggesting that ACOT2 may drive MYCN-mediated tumorigenesis via lipid desaturation, providing a potential target for liver cancer treatment (122). Similarly, in non-small cell lung cancer, ACOT7 promotes cell proliferation by suppressing both apoptosis and ferroptosis. Its expression is directly regulated by the transcription factor ARNTL2, indicating the ARNTL2-ACOT7 axis as a novel potential lung cancer target (62). Furthermore, THEM4 has been shown to mediate the reversal of tamoxifen resistance through ELOVL2: overexpression of ELOVL2 in resistant cells upregulates THEM4, which in turn inhibits the AKT signaling pathway and restores approximately 70% of drug sensitivity (123). In non-cancerous diseases, allosteric inhibitors that selectively bind to the START domain of ACOT11 have been identified via high-throughput screening; these compounds promote fatty acid oxidation and reduce glucose production in brown adipocytes and hepatocytes. Although currently being developed for non-alcoholic fatty liver disease, their mechanism of action suggests that allosteric inhibition of ACOT11 could reshape intracellular lipid metabolic flux (124).

Traditional Chinese medicine-derived compounds have also demonstrated antitumor effects through targeting ACOT family members. For example, piperine from *Piper nigrum* inhibits ACOT1, thereby cutting off the “fuel” supply to gastric cancer cells and suppressing tumor growth (125), while rhodonol inhibits gastric cancer progression by disrupting fatty acid metabolism via miR-1343-3p-mediated inhibition of ACOT11 (126).

#### 4.2.2 The potential of ACOTs as targets for chemotherapy sensitization

ACOTs have been shown to influence the sensitivity of various tumor cells to chemotherapy and radiation therapy. For example, Low expression of ACOT7 enhances the sensitivity of breast and lung cancer cells to DNA-damaging agents, including ionizing radiation (IR) and doxorubicin (Doxo). ACOT7 siRNA transfection alone significantly inhibits cancer cell proliferation by activating the PKCζ (Protein kinase C zeta)–p53–p21 signaling pathway; combination therapy with ACOT7 siRNA and IR or Doxo further amplifies this pathway, enhancing the sensitivity of cancer cells to these DNA-damaging agents. Depletion of ACOT7, together with the acyl-CoA thiolase inhibitor NDGA, induces cell cycle arrest through p53–p21 accumulation (127).

In non-small cell lung cancer, ACOT7 promotes cisplatin resistance by suppressing ferroptosis. Mechanistically, the deubiquitinating enzyme USP3 stabilizes ACOT7 protein levels by removing its ubiquitin modifications; the stabilized ACOT7 then reduces fatty acid peroxidation and inhibits ferroptosis, thereby enhancing tumor cell resistance to cisplatin (29). In retinoblastoma (RB), ACOT7 is upregulated in vincristine (VCR)-resistant cells, where its overexpression drives fatty acid metabolism and autophagy to promote chemotherapy resistance. Treatment with orlistat, a lipase inhibitor that acts as an ACOT7 inhibitor in this context, reduces fatty acid metabolism, cell viability, autophagy, and epithelial-mesenchymal transition (EMT), while increasing RB-resistant cell sensitivity to VCR (128).

In pancreatic ductal adenocarcinoma (PDAC), ACOT8 is significantly overexpressed in gemcitabine-resistant tissues and correlates with poor patient prognosis. Mechanistic studies indicate that ACOT8 suppresses ferroptosis and drives gemcitabine resistance by regulating cholesterol ester levels, reducing polyunsaturated fatty acid-bound phosphatidylethanolamine species, and activating peroxisomes. Knockdown of ACOT8 reactivates ferroptosis-related pathways and enhances tumor cell sensitivity to gemcitabine. Notably, orlistat also inhibits ACOT8, reversing the ferroptosis suppression caused by ACOT8 overexpression. When combined with gemcitabine in PDAC organoids and mouse models, orlistat significantly suppresses tumor growth, suggesting that orlistat combined with gemcitabine is a potential strategy to overcome chemotherapy resistance in pancreatic cancer (62). Similar findings support the use of orlistat in hepatocellular carcinoma (HCC), where it exhibits antitumor activity and enhances paclitaxel sensitivity. Mechanistically, orlistat suppresses the expression of key fatty acid metabolism enzymes, including ACOT8 and FASN, thereby blocking lipid synthesis, inducing G0/G1 cell cycle arrest, inhibiting proliferation, and promoting apoptosis in HCC cells (129). In addition to these *in vitro* observations, *in vivo* studies using murine xenograft models have demonstrated the antitumor activity of orlistat in other tumor types. In prostate cancer xenografts, orlistat combined with radiotherapy significantly suppressed tumor growth (130), and in colorectal carcinoma-bearing mice, orlistat treatment resulted in approximately 55% tumor growth inhibition (131). These findings collectively support the translational potential of orlistat as an antitumor agent.

#### 4.2.3 Prospects for metabolic-immune combination strategies targeting ACOTs

Given the regulatory roles of ACOTs and their derived lipid mediators in metabolic reprogramming and the TME, metabolic-immune combination strategies targeting ACOTs hold promise for clinical translation and warrant further investigation. Preclinical studies support the combination of ACOT inhibitors with chemotherapy; however, no direct reports have yet described their combination with immune checkpoint inhibitors. Nonetheless, emerging evidence indicates that expression levels of several ACOT family members correlate with tumor immune infiltration and response to immunotherapy (55, 72, 132), suggesting that ACOT may serve as a potential therapeutic target or biomarker for immunotherapy.

**Figure 6:**
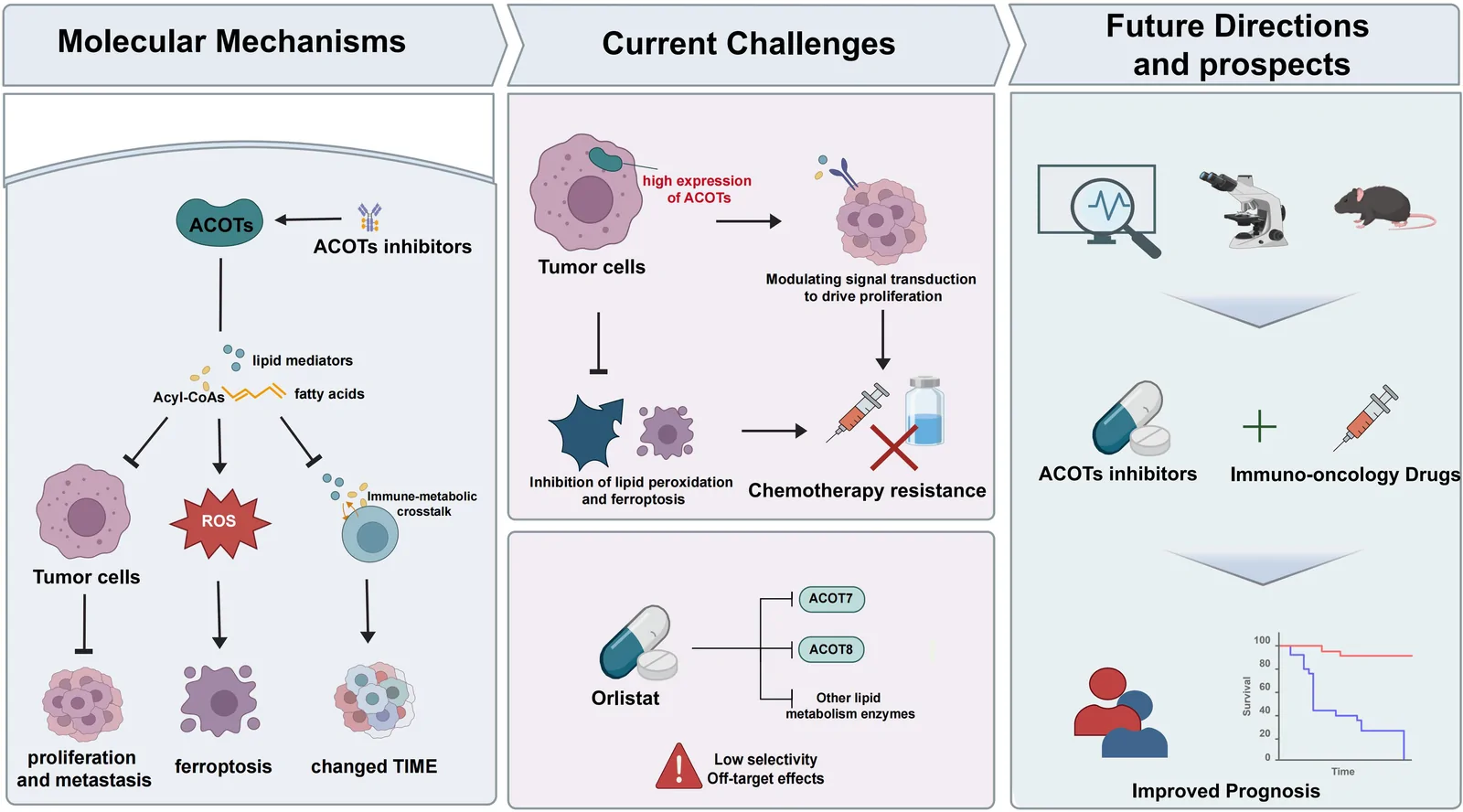
ACOTs promote tumor progression and chemotherapy resistance by suppressing ferroptosis and remodeling the tumor immune microenvironment. Current challenges include high ACOT expression and the low selectivity of Orlistat. Future directions focus on developing selective ACOT inhibitors for combination with immunotherapy. Created in BioRender. Ren. (2026) https://BioRender.com/4ga8wzh.

In terms of drug development, orlistat—a marketed inhibitor of ACOT8 and ACOT7 with a favorable safety profile—offers a promising opportunity for drug repurposing. However, orlistat lacks high selectivity for ACOT8 and ACOT7, as it also inhibits multiple other fatty acid metabolic enzymes, raising concerns about potential off-target effects that require careful evaluation (133) (**Figure 6**).

These limitations highlight the urgent need for better pharmacological tools and a clearer understanding of isoform-specific functions. The ACOT family comprises 12 members with distinct substrate preferences, subcellular localizations, and tissue distribution patterns. Which isoforms are most relevant for TME remodeling and immunotherapy response remains unclear. Adding to this complexity, currently available tool compounds—such as orlistat—lack the selectivity needed to distinguish ACOT-mediated effects from off-target activities. Therefore, future efforts should focus on developing isoform-selective ACOT inhibitors through structure-based design and high-throughput screening. Equally important is the systematic evaluation of these inhibitors in a cell-type- and context-dependent manner, particularly to distinguish tumor cell-autonomous effects from those mediated through immune cells. Such investigations are essential to validate ACOTs as therapeutic targets and to design rational combination strategies with immunotherapy.

## 5 Discussion

The ACOTs act as “hydrolysis nodes” within the acyl-CoA metabolic network, generating lipid mediators that facilitate metabolic and immune regulatory feedback loops through their substrate specificity, subcellular localization, and regulation. Furthermore, ACOT proteins also respond to microenvironmental signals via non-canonical mechanisms—such as transcriptional regulation, RNA modification, and protein phosphorylation—to promote tumor progression.

Currently, no drugs directly targeting ACOT enzymes have reached clinical trials. However, evidence from studies on related enzymes within the acyl-CoA metabolic network suggests that ACOT may be a promising candidate for future drug development, particularly in combination with chemotherapy and immune checkpoint inhibitors. Despite these promising perspectives, several critical gaps and limitations must be acknowledged.

First, although we have systematically compiled the available evidence, the field is still nascent. Most of the current data linking ACOTs to the TME are correlational rather than causal. The literature largely consists of pan-cancer analyses, transcriptomic correlations, and prognostic associations—observations that suggest connections between ACOTs and immune parameters, but do not establish causality. Direct genetic or pharmacological intervention studies are scarce. A case in point: ACOT7 was identified as one of eight lipid metabolism genes associated with CD8^+^ T cell exhaustion in HCC through transcriptomic analysis (134). This finding is suggestive, but it does not tell us whether ACOT7 actively drives T cell dysfunction or merely correlates with it. Nor does it clarify whether the relevant site of action is in tumor cells, immune cells, or both. Without cell-type-specific loss- or gain-of-function models, the causal direction remains unresolved.

Second, the functional heterogeneity among ACOT family members has not been fully characterized. Most studies have focused on ACOT7 and ACOT8, leaving other members such as ACOT6, ACOT13, and THEM5 largely unexplored in the tumor context. This selective focus creates a potential bias: the current understanding of “ACOT function” may not be generalizable across the family. Given that these unstudied members have distinct substrate preferences and subcellular localizations, their roles in tumor metabolism and immunity could be fundamentally different. This selective research focus may bias our understanding of the ACOT family as a whole.

Third, no isoform-selective ACOT inhibitors have been reported. Orlistat, the most widely used compound reported to inhibit ACOT7 and ACOT8, lacks selectivity and also targets multiple other fatty acid metabolic enzymes. Consequently, pharmacological studies using orlistat cannot be unequivocally attributed to ACOT inhibition, leaving a critical gap in functional validation and target prioritization.

Another major gap concerns the cell-type-specific functions of ACOTs. Most studies have focused on ACOT expression in tumor cells, whereas their roles in immune cells—such as T cells, macrophages, and dendritic cells—remain largely unexplored. Given that acyl-CoA metabolism shapes immune cell differentiation and effector functions, it is plausible that ACOTs directly modulate immune cell fate through intracellular acyl-CoA pools. This possibility warrants systematic investigation using immune-cell-specific knockout models.

Future research should address several key challenges: elucidating the functional heterogeneity and the lipid metabolic reprogramming mediated by ACOT members across different tumor types and TME; developing selective ACOT inhibitors with systematic evaluation of efficacy, safety, and immune effects; and translating preclinical evidence of synergistic effects with chemotherapy and immunotherapy into clinical applications.
